# Potent Impact of Plastic Nanomaterials and Micromaterials on the Food Chain and Human Health

**DOI:** 10.3390/ijms21051727

**Published:** 2020-03-03

**Authors:** Yung-Li Wang, Yu-Hsuan Lee, I-Jen Chiu, Yuh-Feng Lin, Hui-Wen Chiu

**Affiliations:** 1 Graduate Institute of Clinical Medicine, College of Medicine, Taipei Medical University, Taipei 11031, Taiwan; 2 Department of Cosmeceutics, China Medical University, Taichung 40402, Taiwan; 3 Division of Nephrology, Department of Internal Medicine, Shuang Ho Hospital, Taipei Medical University, New Taipei City 23561, Taiwan

**Keywords:** plastic products, food chain, microplastics, nanoplastics

## Abstract

Plastic products are inexpensive, convenient, and are have many applications in daily life. We overuse plastic-related products and ineffectively recycle plastic that is difficult to degrade. Plastic debris can be fragmented into smaller pieces by many physical and chemical processes. Plastic debris that is fragmented into microplastics or nanoplastics has unclear effects on organismal systems. Recently, this debris was shown to affect biota and to be gradually spreading through the food chain. In addition, studies have indicated that workers in plastic-related industries develop many kinds of cancer because of chronic exposure to high levels of airborne microplastics. Microplastics and nanoplastics are everywhere now, contaminating our water, air, and food chain. In this review, we introduce a classification of plastic polymers, define microplastics and nanoplastics, identify plastics that contaminate food, describe the damage and diseases caused by microplastics and nanoplastics, and the molecular and cellular mechanisms of this damage and disease as well as solutions for their amelioration. Thus, we expect to contribute to the understanding of the effects of microplastics and nanoplastics on cellular and molecular mechanisms and the ways that the uptake of microplastics and nanoplastics are potentially dangerous to our biota. After understanding the issues, we can focus on how to handle the problems caused by plastic overuse.

## 1. **Introduction**

**Figure 1:**
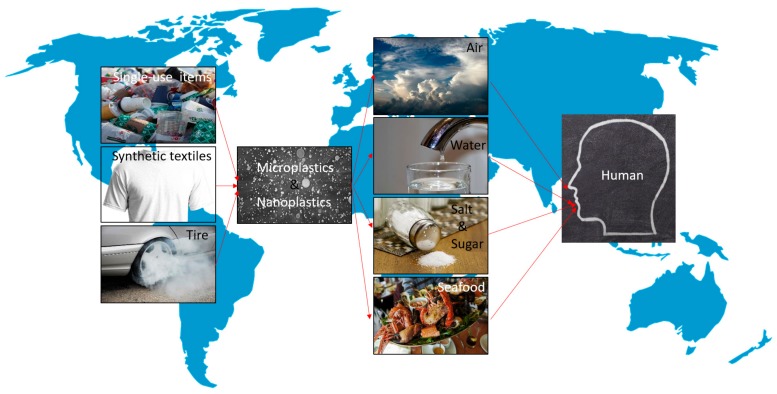
Some sources and deposits of microplastic and nanoplastic are the result of human needs. The potential impacts of these plastics on air, water, and many foods finally returns to affect humans. All pictures come from Pixabay (https://pixabay.com/).

Recently, plastic products have become inexpensive and convenient and are used in all aspects of daily life, such as food and product packaging, clothing, construction and car materials, household goods, medical devices, personal care products, and, toys. [1,2]. Although plastic products are relatively convenient, the negative influences of the “plastic era” caused by these inexpensive and convenient products include high levels of plastic production coupled with a slow biodegradation rate, uncontrolled use, and ineffective and irresponsible waste recycling, leading to plastic accumulation in our global environment, particularly in freshwater and marine environments [3,4,5,6,7,8,9,10]. The use of plastic products has increased rapidly, and 33 billion tons of plastic will likely be produced by 2050 [11], making the Pacific Ocean a giant garbage dump [12]. The plastic debris in aquatic environments is fragmented into smaller pieces by ultraviolet light and biodegraded plastic forms microplastics and nanoplastics [13,14,15]. However, the largest proportion of microplastics and nanoplastics is generated from the laundering of textiles with mixed synthetic fibers [16] and the friction of the tires of moving cars [17,18]. These microplastics and nanoplastics have unclear effects on organismal systems. Recently, evidence has been presented indicating that plastics significantly affect the growth and oxygen production of *Prochlorococcus* and microalgae. *Prochlorococcus,* especially, is the ocean’s most abundant photosynthetic bacteria and produces 10% of global oxygen. [19,20]. However, the growth of earthworms is meaningfully different in soil ecosystems, particularly agricultural land, contaminated with microplastics [21]. In addition, it is noteworthy that microplastics are widespread in naturally-occurring Arctic deep-sea sediments [22] and in snow ranging from the Alps to the Arctic [23]. Therefore, microplastic and nanoplastic contamination is everywhere [24]. Microplastics have been found in human stool [25] and humans can consume microplastics and nanoplastics through seafood [26,27,28,29,30] and water [31,32,33,34,35,36], etc. Whether plastics will harm our health is unclear; however, the potential consequences may affect the ecological functioning of the globe and future generations of organisms (Figure 1). In this review, we briefly introduce a classification of plastic materials and describe the origin of microplastics and nanoplastics, the food contaminated by microplastics and nanoplastics, the damage and diseases caused by microplastics and nanoplastics, the molecular and cellular mechanisms of the damage and diseases caused by microplastics and nanoplastics, and solutions to mediate the problems caused by plastic overuse.

## 2. **Classification of Plastic Materials and Related Product Applications**

**Figure 2:**
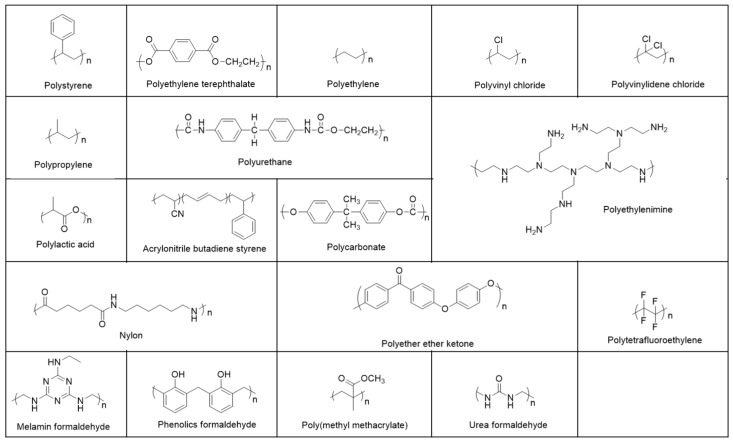
Typical polymer types and their chemical structures. Chemical structures are shown for polyethylene (PE), polyester (PES), polyethylene terephthalate (PET), polyethylenimine (PEI) (Ultem), polystyrene (PS), polylactic acid (PLA), polypropylene (PP), polyvinyl chloride (PVC), polyvinylidene chloride (PVDC) (Saran), polycarbonate (PC), polycarbonate/acrylonitrile butadiene styrene (PC/ABS), polyamides (PA) (nylon), acrylonitrile butadiene styrene (ABS), polyurethanes (PU), urea–formaldehyde (UF), melamine formaldehyde (MF), polymethyl methacrylate (PMMA), and polytetrafluoroethylene (PTFE).

Plastic production has gradually increased every year, from 1.5 million tons in the 1950s [37] to an estimated 33 billion tons in 2050 [11]. Plastics are specifically derived from synthetic polymers generated by the polymerization of many monomers and mixtures of a range of materials [38]. Therefore, plastic is predominantly generated into polyethylene (PE), polyester (PES), polyethylene terephthalate (PET), polyetherimide (PEI) (Ultem), polystyrene (PS), polypropylene (PP), low-density polyethylene (LDPE) high-density polyethylene (HDPE), polyvinyl chloride (PVC), polyvinylidene chloride (PVDC) (Saran), polycarbonate (PC), polycarbonate/acrylonitrile butadiene styrene (PC/ABS), high-impact polystyrene (HIPS), polyamides (PA) (nylon), acrylonitrile butadiene styrene (ABS), polyurethanes (PU), urea–formaldehyde (UF), melamine formaldehyde (MF), polymethyl methacrylate (PMMA), polytetrafluoroethylene (PTFE), and polylactic acid (PLA), etc. [39] (Figure 2). The highest percentages of plastics produced worldwide meet the definition of thermoplastic: PP (21%), LDPE (18%), PVC (17%), and HDPE (15%) [40]. Plastic polymers have numerous applications in daily life [41]. PP is usually used in pots for plants, bags, industrial fibers, netting, medical masks, bottle caps, ropes, straws, containers, tanks and jugs, appliances, car fenders, plastic pressure pipe systems, and centrifuge tubes. LDPE is usually used in outdoor furniture, siding, wire cable, floor tiles, plastic bags, shower curtains, buckets, clamshell packaging, and soap dispenser bottles. PVC is usually used in plumbing pipes and guttering, siding, shower curtains, blood bags, window frames, and flooring. HDPE is usually used in detergent bottles, plastic bottles, plastic bags, bottle caps, and milk jugs [37,39,42].

## 3. **Routes of Plastic Micromaterial and Nanomaterial Pollution**

Plastic fragments can be generally divided into several types: macroplastics and mesoplastics are greater than 5 mm in size [43], microplastic particles are smaller than 5 mm [44], and nanoplastics are less than 1000 nm or 100 nm [45]. Currently, microplastics are found worldwide in freshwater and marine systems [46], in sediment [47], in soil [48], and within biota [49]. Nanoplastics are generated by the abiotic and biotic degradation of microplastics. For example, UV degradation of microplastics has been shown to generate nanoplastics [13,50], and digestive fragmentation has been proposed as a means by which nanoplastics can be generated from microplastics [51]. However, two types of microplastics, primary and secondary microplastics, are categorized by the form in which they are released. Primary microplastics are directly released into the environment as small particles. Secondary microplastics are derived from large plastic items being degraded into small plastic fragments upon exposure to the environment [52]. The largest proportion of these particles is derived from laundering textiles with mixed synthetic fibers [16] and the friction of car tires [17,18]. Secondary microplastics from synthetic textiles in garments are the major type of microplastics [53,54]. Each textile may release approximately 1900 fibers per washing [55]. Other sources of microplastics and nanoplastics are urban dust [56], road markings [17], and personal care products [57].

## 4. **Presence of Plastic Micromaterials and Nanomaterials in Food** **and Food Products**

Microplastics and nanoplastics are currently everywhere. In marine environments, seabirds and marine mammals ingest microplastics at low trophic levels [52,58]. Microplastics and nanoplastics have been detected at the base of the food web, specifically, in zooplankton, such as chaetognaths [59]. Crustaceans, such as the Japanese shore crab [60] and North Pacific krill [61], contain microplastics and nanoplastics. Sea fish, such as the northeastern Pacific Ocean forage fishes [62], areolate grouper and goldbanded jobfish, are contaminated with microplastics [63]. Oysters ingest polystyrene microplastics, which affect their reproduction [64]. The mussel *Mytilus edulis* ingests microplastics that translocate to the circulatory system [65]. Many kinds of mussels are contaminated, including blue mussels [26,37,66], Mediterranean mussels [61,67], brown mussels [66], and northern horse mussels [68]. Plastic additives and hydrophobic organic compounds (HOCs) are also found in mussels [69]. In summary, many kinds of seafood are potentially contaminated by microplastics and/or nanoplastics [30]. In addition, in our daily life, many consumables, such as tap water [70], bottled water [34,71], beer [70,72], sea salt [70,73], sugar [74], honey [74] and plastic teabags [75] have also been found to contain microplastics or nanoplastics. Even air [76,77] and unprocessed water [78] have been contaminated with microplastics. Sooner or later, the entire food chain will be contaminated with plastic. Some statistics from studies published by PubMed on animals contaminated by microplastics and/or nanoplastics are presented in Table 1.

## 5. Damage and Diseases Caused by Plastic Micromaterials and Nanomaterials

Many marine animals suffer from ingesting high amounts of plastic debris [79,80]. Fragments of this plastic debris, such as microplastics, accumulate in the gut and cause obstruction and inflammation in many organs in a wide range of living creatures [52,81]. Microplastics reduce photosynthesis in microalgae [20] and have a negative influence on the feeding behavior of zooplankton [58] and lugworms [82]. They also accumulate in and probably negatively influence the gills, stomach, and hepatopancreas of crabs [83], and they change the biomarkers and histology of fish tissues [84]. Evidence indicates that PS microplastics decrease the number of eggs and larvae produced and sperm velocity of oysters [64]. PS microplastics may also transport contaminants to microorganisms [81]. Studies have described the influence of microplastics on the digestive system [85]. The aquatic ecosystem has accommodated the ingestion of contaminated organisms [86]. Finally, this leads to the uptake of microplastics in the human intestine [26]. Several studies have indicated that PS microplastics can cause metabolic disorders of amino acids, bile acids [87], and liver lipids [88] in mice. Microplastics change gut microbiota dysbiosis and decrease gut mucin secretion in mice [87,88]. However, these microplastics or nanoplastics are also released to the atmosphere, becoming airborne contaminants [55,86,89]. Indeed, a study shows contamination in working environments [33]. Workers in the synthetic textiles, flock and vinyl chloride (VC), or polyvinyl chloride (PVC) industries are potentially exposed to high concentrations of microplastics in the air during work [76]. Synthetic textile workers potentially suffer higher rates of lung-cancer-related mortality [90] or stomach and esophageal cancers [91]. Flock workers have a high incidence of interstitial and lung diseases [92,93]. VC has been considered a carcinogenic factor and mostly causes angiosarcoma of the liver [94,95,96]. Microplastics or nanoplastics disrupt the endocrine system [97], induce neurotoxicity [98], and produce reproductive abnormities with trans-generational effects [99,100,101,102,103]. In addition, food and drink are a major vehicles of microplastic and nanoplastic exposure through which polymer elements and additives are potentially transported [104]. Risk assessments on using food packaging nanomaterials with antimicrobial activity, including titanium dioxide [105] and carbon nanotubes [106], have shown that they present risks comparable to those of using nanopolymers. The complex mixtures of plastic additives can dissolve in the polymer and leak into the surrounding environment [107]. The physical–chemical characteristics of these particles, such as the size, external charge, length:width ratio, porosity, surface corona, and hydrophilicity, cause different circulation times [108]. In addition, microplastics can absorb persistent organic pollutants (POPs) such as polycyclic aromatic hydrocarbons (PAHs), polychlorinated biphenyls (PCBs), and pesticides, including dichlorodiphenyltrichloroethane (DDT) and hexachlorobenzene (HCB), in the ocean [109,110]. These compounds have a higher affinity for plastic than for water [110,111]. Microplastics and/or nanoplastics are taken up into the gut and lungs, and enter many organs, where they potentially cause damage and result in disease.

## 6. Molecular and Cellular Mechanisms of Plastic Micromaterial and Nanomaterial Damage and Disease

**Figure 3:**
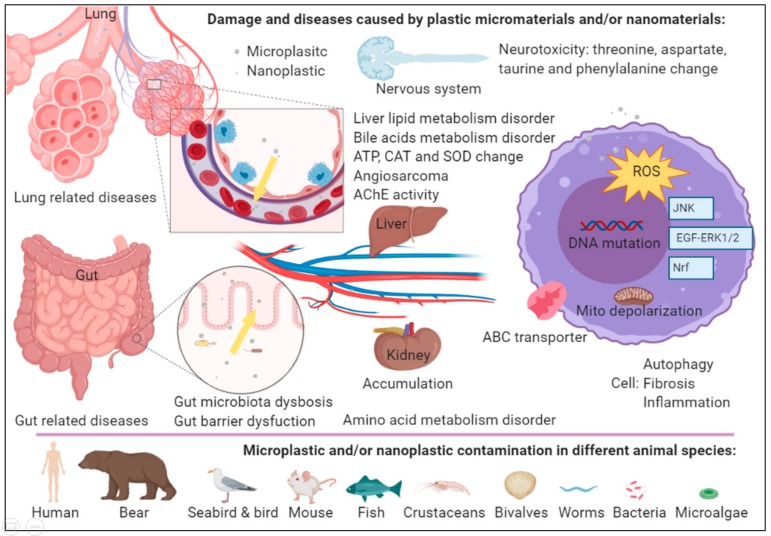
Impact of plastic micromaterials and nanomaterials in organisms. Microplastics and/or nanoplastics can enter the circulation from the gut and lungs and accumulate in the gut, liver, and kidney resulting in several diseases. At the cell level, microplastics or nanoplastics can inhibit the efflux pump and mitochondria depolarization, induce reactive oxygen species (ROS). They also affect several signaling pathways, cause fibrosis, autophagy, and even DNA mutations. Many animal species have been contaminated by microplastics and/or nanoplastics. The figure was created with BioRender.com.

Microplastics and/or nanoplastics can enter the circulation from the gut via trophic transfer [30] and from air [76,77]. Microplastics or nanoplastics inhibit the efflux pump and induce cytotoxicity in human intestinal cells [112]. The cytotoxicity induced by microplastics and/or nanoplastics stimulates oxidative stress via free radical generation originating from reactive oxygen species (ROS) [98,101,103,113,114,115]. Several studies have shown this connection in monogonont rotifer [116], *Caenorhabditis elegans* [117], *Danio rerio* [118], mouse liver [119], and human intestine cell lines [112]. Overproduced ROS can alter the homeostasis of cells by mediating antioxidant systems. ROS overwhelm the antioxidants produced in response to damage to cellular components, including DNA, carbohydrates, lipids, and proteins. This damage is associated with gene instability, physiological alterations, and carcinogenesis [120,121]. For example, scleractinian coral, *Pocillopora damicornis*, exposed to microplastics have increased superoxide dismutase (SOD) and catalase (CAT) activity and glutathione S-transferase (GST) and alkaline phosphatase (ALP) loss of function. SOD and CAT are antioxidant enzymes, GST is a detoxifying enzyme and ALP is an immune enzyme in coral. In addition, in coral, microplastics regulate genes that are related to the stress response, zymogen granules, c-Jun N-terminal kinase (JNK) signaling pathways, sterol transport, and the epidermal growth factor–extracellular signal-regulated kinase 1/2 (EGF-ERK1/2) pathway (Figure 3) [115]. In addition, PS nanoplastics increase oxidative stress, activate the expression of genes in the nuclear factor E2-related factor (Nrf) signaling pathway (Figure 3) [114], and increase expression of glutathione S-transferase 4 (GST-4) enzyme in *Caenorhabditis elegans* [117]. Additionally, a previous report showed that PS microplastics also induce inflammation and activate SOD and CAT activity in the livers of *Danio rerio* [118] and mice [119,122]. These findings indicate that microplastics induce oxidative stress as the main mechanism of toxicity induction in these organisms. PS microplastics can affect amino acid metabolism by increasing arginine and tyrosine and affect bile acid metabolism by mediating the levels of taurocholic acid (TCA), β-muricholic acid (βMCA), adenosine triphosphate (ATP)-binding cassette, subfamily B, member 11 (*Abcb11*) and cholesterol 7a-hydroxylase (*Cyp7a1*) [87]. They also affect liver lipid metabolism by changing the triglyceride (TG), total cholesterol (TCH), and pyruvate levels (Figure 3) [88]. PS microplastics also increased the acetylcholinesterase (AChE) activity and the related neurotransmitters such as threonine, aspartate, and taurine in a mouse model [122]. In addition, microplastics and nanoplastics elicit immunological responses [115,123,124], alter gene expression [88,103,113,114,125] and induce genotoxicity [113,126]. In kidney cells, VC stimulates the expression of fibrosis-related proteins, such as CTGF, PAI-1, and collagen 1, and autophagy-related proteins, such as Beclin 1 and LC3-II [127]. VC is also a carcinogenic factor and results in angiosarcoma of the liver [94,95,96]. Studies have indicated that VC causes several DNA mutations, such as *Ras* mutations [128], *K-ras-2* mutations [129], *p53* mutations [130,131], and *p21* mutations [132].

## 7. Solutions for Reducing Plastic Micromaterials and Nanomaterials

A large area of accumulated garbage is adrift in the ocean [133]. Prevention and clean-up proposals have been made by political bodies around the world [134], such as the plastic reduction policy of Africa, which ranks first in the world [135]. A Netherlands-based organization, the Ocean Cleanup, uses massive drift nets to reduce the size of the Great Pacific Garbage Patch [136]. Wastewater treatment plants (WWTPs) in several countries have found microplastic particles [49,137,138,139]. Australia uses filters in large drains to stop garbage from entering the ocean [140]. Single-use plastic items are one of the components in this large area of plastic waste. In India, single-use plastic items, such as plastic bags, plastic spoons, plastic cups, plastic drinking straws, plastic jars, and plastic bottles, have been banned since October 2, 2019. The European Union has set a target to eliminate some single-use plastic items by 2021 [141]. Single-use plastic items, such as plastic straws, are being replaced⁠—a Vietnamese company has developed a reed pipe to replace plastic straws [142], and a Taiwanese company has developed a straw with sugar cane [143]. In addition, facial cleansers containing plastic particles [57] have been banned in many countries [144]. On the one hand, plastic waste has been turned into resources. For example, a company in the Netherlands uses plastic to replace traditional road materials [145], and it is better than asphalt, with 60% greater strength [146]. In India, abandoned fishing nets have been turned into surfboards [147], in UK, students have successfully used fish skin and red algae as raw materials to develop plastic substitutes [148], and in Mexico, scientists have used cactus fruit to make nontoxic edible plastic [149]. On the other hand, due to physical and chemical changes, plastics become microplastics and nanoplastics. Therefore, some microbial biodegradation can be used to depolymerize those polymers into smaller monomers. Biodegradation is ultimately successful when plastics degrade monomers into CO_2_ and water. Marine bacteria are potential candidates for use in the biodegradation of plastic wastes [150]. PS is known to biodegrade in the gut of yellow mealworms [151]. Many fungal strains can also degrade several plastics, such as PVC, PHB, and PLA [39]. Recently, several enzymes have been identified as capable of degrading PET plastics [152].

## 8. Conclusions

We over-use plastics because they are inexpensive and convenient, and worldwide, ecosystems are suffering, and contaminating-levels of plastic debris is a concern that has been reported. Plastic must be managed (especially in single-use items) and recycled such that it is finally fragmented to small plastics. Most of the contamination by microplastics and nanoplastics is derived from laundering synthetic textiles and the friction from the tires of driven cars. Currently, there is no effective way to reduce the amount of microplastics and nanoplastics in the food chain. Furthermore, it is unclear how the mixture of different sized groups and material types interacts with living creatures. Previous studies have indicated that workers in plastic-related industries suffer many kinds of cancer by being exposed to high levels of airborne microplastics over many years. In addition, it is important to characterize the microplastics and nanoplastics that have accumulated in the food chain and to gain a clearer understanding of their negative impact on our bodies. Finally, the degradation of microplastics and nanoparticles from environmental bacteria and fungi remains a challenge for the scientific community.

## Figures and Tables

**Table 1:** Published studies from the National Center for Biotechnology Information (NCBI) on microplastic and/or nanoplastic contamination in different animal species.

Animal Species	Number of Published Studies from NCBI
Human	2
Bear Mouse	1 5
Birds	5
Seabirds	8
Crustaceans	68
Bivalves	79
Fish and sea mammals	161
Insects	5
Turtles	5
Amphipods	2
Seaplants	3
